# Automated selection of nanoparticle models for small-angle X-ray scattering data analysis using machine learning

**DOI:** 10.1107/S2053273324000950

**Published:** 2024-02-29

**Authors:** Nicolas Monge, Alexis Deschamps, Massih-Reza Amini

**Affiliations:** a Xenocs, Grenoble, France; b SIMaP, University of Grenoble Alpes, CNRS, Grenoble INP, Grenoble, France; c LIG, University of Grenoble Alpes, CNRS, Grenoble, France; Deutsches Electronen-Synchrotron, Germany

**Keywords:** machine learning, nanoparticles, SAXS, small-angle X-ray scattering, data analysis, model selection

## Abstract

Small-angle X-ray scattering (SAXS) is widely used to analyze the shape and size of nanoparticles in solution. A multitude of models, describing the SAXS intensity resulting from nanoparticles of various shapes, have been developed by the scientific community and are used for data analysis. Choosing the optimal model is a crucial step in data analysis, which can be difficult and time-consuming, especially for non-expert users. An algorithm is proposed, based on machine learning, representation learning and SAXS-specific preprocessing methods, which instantly selects the nanoparticle model best suited to describe SAXS data. The different algorithms compared are trained and evaluated on a simulated database. This database includes 75 000 scattering spectra from nine nanoparticle models, and realistically simulates two distinct device configurations. It will be made freely available to serve as a basis of comparison for future work. Deploying a universal solution for automatic nanoparticle model selection is a challenge made more difficult by the diversity of SAXS instruments and their flexible settings. The poor transferability of classification rules learned on one device configuration to another is highlighted. It is shown that training on several device configurations enables the algorithm to be generalized, without degrading performance compared with configuration-specific training. Finally, the classification algorithm is evaluated on a real data set obtained by performing SAXS experiments on nanoparticles for each of the instrumental configurations, which have been characterized by transmission electron microscopy. This data set, although very limited, allows estimation of the transferability of the classification rules learned on simulated data to real data.

## Abstract 2

Many models have been developed for analyzing SAXS data; however choosing the optimal model is difficult and time-consuming, especially for non-expert users. This paper proposes an algorithm, based on machine learning, representation learning and SAXS-specific preprocessing methods, which instantly selects the nanoparticle model best suited to describe SAXS data.

## 1. Introduction

Small-angle scattering (SAS) methods are among the most useful tools for analyzing the internal structure of materials at the nanometre scale. One of the strengths of the SAS techniques lies in the broad range of use. Particles with a wide size range from 1 to 100 nm can be analyzed (Allec *et al.*, 2015 ▸), and one can obtain structural information about their shape, size and electronic density (Tobler *et al.*, 2009 ▸; Talapin & Shevchenko, 2016 ▸). This adaptability makes SAS methods increasingly popular for analysis of metals (De Geuser & Deschamps, 2012 ▸; Li *et al.*, 2016 ▸), polymers (Portale *et al.*, 2013 ▸), surfaces (Miranda *et al.*, 2014 ▸) or biomolecules (Da Vela & Svergun, 2020 ▸). One of SAS’s drawbacks is that the experiments only provide an indirect characterization, whose interpretation requires the use of appropriate models, necessitating choices and a high level of expertise. Once an interpretation model is chosen, software tools exist to refine the model parameters on the experimental data. For instance, *SASView* (https://www.sasview.org/) is a free open-source program that includes tools for analyzing and fitting 200 SAS models. Thus, the difficulty consists of choosing among the many available models, and a newcomer can be easily overwhelmed by the amount of tunable parameters. One popular strategy is to choose a few alternative models that may fit the data well, then use a regression procedure like expectation–maximization (Bakry *et al.*, 2019 ▸; Moon, 1996 ▸; Benvenuto *et al.*, 2016 ▸) or a Gaussian process (Fong *et al.*, 2021 ▸) to choose the best fit. Because this is an ill-posed problem, several models and parameters may produce similar results; hence regression-based techniques rely largely on the initialization step. As a result, prior knowledge and competence with SAS data processing are required in order to achieve appropriate model selection prior to regression approaches.

Several studies have been carried out in order to overcome this problem, especially using machine learning techniques to assist users in the selection of the best model for analyzing small-angle X-ray scattering (SAXS) data (Franke *et al.*, 2018 ▸; Archibald *et al.*, 2020 ▸; Tomaszewski *et al.*, 2021 ▸), small-angle neutron scattering (SANS) data (Do *et al.*, 2020 ▸; Tung *et al.*, 2022 ▸), and deep learning has been used for characterization of SAXS (Molodenskiy *et al.*, 2022 ▸; Abdel Aty *et al.*, 2022 ▸) and grazing-incidence small-angle X-ray scattering (GISAXS) (Liu *et al.*, 2019 ▸) data.

These machine learning approaches have been shown to be effective in finding relationships between input data and their desired corresponding output. The use of machine learning techniques becomes even more attractive with simulation tools such as *SASView*, which allows one to generate the large number of simulated SAXS data needed for the training of learning algorithms. Recent studies have demonstrated that learning approaches can associate SAXS or SANS curves to the geometric shape or dilute particle structure of the studied material (Archibald *et al.*, 2020 ▸; Do *et al.*, 2020 ▸). However, the outcome of these studies reveals that this classification task remains inaccurate when the models used for the curve generation consider models with similar shapes, like spheres and ellipsoids, as an example. Inter-curve criterion-based approaches, such as K-nearest neighborhoods (KNN) and variations (Archibald *et al.*, 2020 ▸), may thus lose efficiency if the models produce similar curves, especially when the data are noisy. Franke *et al.* (2018 ▸) and Liu *et al.* (2019 ▸) proposed to represent the data in another space than the intensity space. Franke *et al.* (2018 ▸) proposed to apply a drastic dimension reduction down to three features based on the integral of the Kratky representation, to classify models of particles with homogeneous scattering length density. Liu *et al.* (2019 ▸) proposed to learn the data transformation using the famous AlexNet neural network, in order to predict a lattice orientation from GISAXS data, with really good results.

While these studies demonstrate the potential of a data-driven approach for automatic model detection, they are limited in several aspects. In the studies of Archibald *et al.* (2020 ▸), Do *et al.* (2020 ▸), Tomaszewski *et al.* (2021 ▸) and Franke *et al.* (2018 ▸), the simulation models used to produce the data did not take into account the laboratory instrument characteristics, whose smaller brilliance and smaller photon flux when compared with synchrotrons generate important noise levels and whose geometrical characteristics induce data smearing and convolution with the source’s characteristics. In machine learning, consistency between training and test data is a key factor in determining the effectiveness of a model. A discrepancy between simulated training data and real test data is likely to render the classification model unusable. The first three aforementioned studies did not test their algorithms on real data, which makes it impossible to validate the relevance of these classification models to a real-life use case. Franke *et al.* (2018 ▸) evaluated the performance of their algorithm on real synchrotron data, but the classification problem was relatively simple because the data sets did not include inhomogeneous particles.

We propose several improvements to overcome these limitations and advance towards an efficient and user-friendly model selection algorithm. We simulate intensity curves of dilute isotropic aqueous solutions of nanoparticles, using a simulation process adapted to create realistic data, representative of data from laboratory instruments. Two variants of the data set are created, each associated with a specific device configuration. A small data set of real data is also produced using these two device configurations. It is used to estimate the transferability of classification rules learned on simulated data to real data. These data sets, used to benchmark different classification models, are available at https://data.mendeley.com/datasets/b96sw3jffy/1 (Monge, 2023 ▸). We manage to surpass the results of previous studies by using representation learning methods. Representation learning techniques have proven to be extremely effective in many fields such as speech recognition (Hinton *et al.*, 2012 ▸), signal processing (Boulanger-Lewandowski *et al.*, 2012 ▸), computer vision (Veit *et al.*, 2017 ▸) or in natural language processing (Astudillo *et al.*, 2015 ▸). The main idea of representation learning is to transform the input data into a new space, named latent space, which directly captures similarity between data. Knowing the association between the input data and the class label, the transformation from input data to latent vector can be fitted to keep only the relevant information for that specific purpose. Input vector transformation can be carried out using deep learning methods, such as neural networks, trained to apply a transformation that will optimize the classification task. Finally, we look at the influence of the device configuration on classification. Using both real and simulated data sets, in the two device configurations, we establish the transferability of rules learned on one configuration to another, and propose a simple method for reducing the number of classification models needed to implement an efficient classification application.

## 2. Proposed approach

### 2.1. Collected data sets

**Figure 1:**
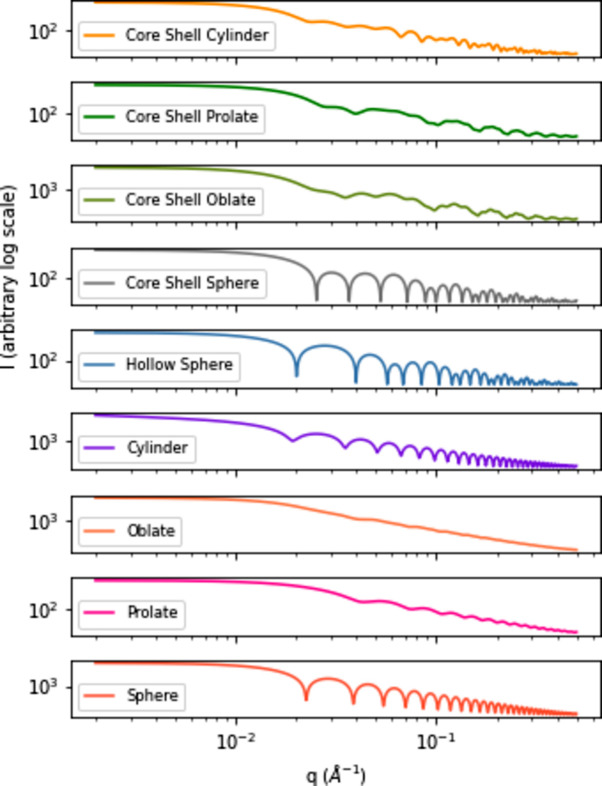
Example of noiseless *I*(*q*) curves generated using the nine form factors, all particle sizes having the same order of magnitude and all particles having the same scattering length density.

Well characterized systematic experimental SAXS data sets covering uniformly the parameter space of form factors are elusive and would be very difficult to obtain. It is therefore complicated to use a database of real SAXS data to train the machine learning algorithms. Fortunately, a large amount of data can be quickly generated using the form factors implemented in *SASView*. In our study, the choice of form factors is voluntarily focused on nine geometric shapes that are representative of classical nanoparticles and can be very similar to each other with a specific choice of parameters, and thus difficult to classify. The form factors used as targets are spheres, cylinders, oblate ellipsoids, prolate ellipsoids and corresponding core shells: core shell spheres (with a dense core), hollow spheres, core shell cylinders, core shell oblates, core shell prolates. This choice is motivated by the desire to push as far as possible the limit of detection of one form factor compared with another, so that a user can choose a form factor as precisely as possible. Also, in order to complete what has been proposed by Tomaszewski *et al.* (2021 ▸) and Franke *et al.* (2018 ▸), a large part of the data set comprises inhomogeneous scattering length density form factors. A comparison of SAXS curves generated with the different form factors is presented in Fig. 1 ▸. The database is composed of 4184 *I*(*q*) curves per form factor, and the range of parameters used for the data generation can be found in Appendix *A*1 (see the supporting information).

In order for the classification model to learn classification rules on synthetic data which are transferable to real data, the synthetic data must be as close as possible to the real data. For this purpose, the ‘perfect’ data generated by *SASView* were processed by Xenocs’ *Xsact* software to add patterns representative of a Xenocs laboratory device to the noiseless data. Two device configurations were simulated:

(i) A Xeuss1800HR configuration, with a Dectris Eiger1M detector, a sample-to-detector distance of 1800 mm, which leads to a 0.0031 to 0.1493 Å^−1^ *q* range, a Cu source with λ = 1.54 Å, an FWHM of the incident beam of 0.0016 Å^−1^, a transmitted flux of 3.43 × 10^6^ photons s^−1^ and a counting time of 20 min. The data set created with this configuration has been used to benchmark the classification model. As the sample-to-detector distance is variable on the Xeuss device, the distance is chosen to suit the size of the nanoparticles to be analyzed.

(ii) A NanoInXiderHR data set, with a Dectris Eiger1M detector, a sample-to-detector distance of 938 mm, which leads to a 0.0019 to 0.4452 Å^−1^ *q* range, a Cu source with λ = 1.54 Å, an FWHM of the incident beam of 0.0024 Å^−1^, a transmitted flux of 7.22 × 10^6^ photons s^−1^ and a counting time of 20 min, which has been used to evaluate the influence of device configuration on classification model performance.

**Figure 2:**
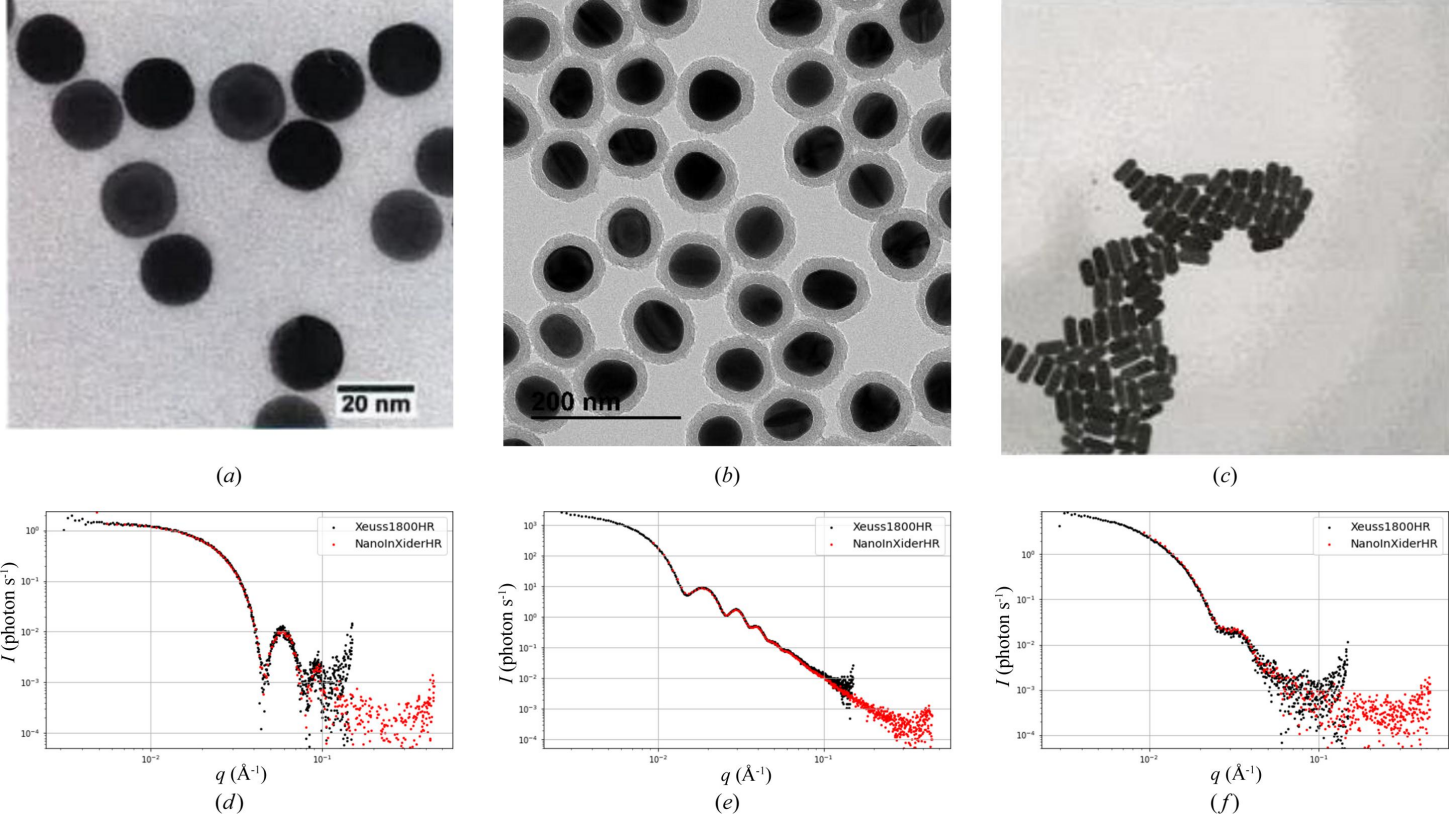
TEM imaging of three nanoparticle samples and corresponding SAXS curves realized with two different devices. Nanoparticles are diluted in solution for the SAXS acquisition, and dried and deposited for TEM imaging, resulting in clustering on the TEM images. (*a*) TEM image of sphere No. 1. (*b*) TEM image of core shell sphere No. 1. (*c*) TEM image of prolate No. 3. (*d*) SAXS curves of sphere No. 1. (*e*) SAXS curves of core shell sphere No. 1. (*f*) SAXS curves of prolate No. 3.

To assess the transferability of classification rules learned on synthetic data to real data, a real data set has been acquired. Ten samples of nanoparticles in solution were characterized by SAXS with the two instrumental configurations cited above, as well as by transmission electron microscopy (TEM) after drying, using a Jeol 1010 instrument working at 100 kV located at CEA/IRAMIS/LIONS, allowing us to determine without ambiguity the form factor, as well as polydispersity, and to check that the aspect ratios of the nanoparticles are well within the limits defined during the simulations. Six samples were labeled as spheres, three as prolate ellipsoids and one as a core shell sphere. The spheres and prolate ellipsoids are Au nanoparticles, the core shell has an Au core and SiO_2_ shell, and nanoparticle diameters ranged from 20 to 150 nm. Fig. 2 ▸ shows three representative examples of TEM images and the associated SAXS curves; TEM images and SAXS curves of all samples are available in Appendix *F*2 (see the supporting information). The experimental SAXS data have been fitted by models corresponding to the observed shapes and to some proposed by the classification model; the resulting fitting parameters are given in Appendix *F*1 (see the supporting information).

### 2.2. Classification model selection

Data preprocessing is a key step in machine learning (Huang *et al.*, 2015 ▸), especially when analyzing SAXS data. A SAXS curve contains structural information, like the size and shape of the analyzed particles. Shape information is located in the central zone of the curve, named the Fourier regime (Boldon *et al.*, 2015 ▸), where amplitude is really weak against the amplitude at small scattering vector *q*, *i.e.* in the Guinier regime where size information is located. A first goal of preprocessing is to weight the amplitudes of the different parts of the curve. For this purpose, two preprocessing steps are proposed in this study: the logarithm (LOG) and the standardization (STD). Let us note:

*q* = [*q*_0_, …, *q*_*j*_, …, *q*_*J*_] is the list of the scattering vector values.

*I*_*n*_(*q*) = [*I*_*n*_(*q*_0_), …, *I*_*n*_(*q*_*j*_), …, *I*_*n*_(*q*_*J*_)] is the *n*th simulated curve, where *I*_*n*_(*q*_*j*_) is the intensity of the *n*th simulation at the *j*th scattering vector value. 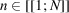 with *N* the total number of simulations in a data set.


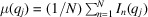
 is the mean of the *j*th intensity values over all the simulations.


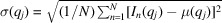
 is the standard deviation of the *j*th intensity values over all the simulations.

Then logarithm preprocessing and standardization are defined as: logarithm (LOG): *I*_*n*_(*q*_*j*_) → log[*I*_*n*_(*q*_*j*_)]; standardization (STD): 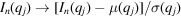 .

In the context of particle shape classification, it is useful to normalize the data against some of the other parameters, such as electronic contrast or volume fraction. Therefore the quantity 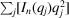 (Guinier *et al.*, 1955 ▸) can be used as a normalization factor. This integral normalization will be noted IntN: 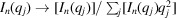 .

In practice, it is common to observe the curves with the *q* axis in a logarithmic scale. The advantage of this practice is that it allows information at medium and high *q* to be visually condensed. Inspired by this practice, a preprocessing designed to condense information in medium and high *q* is proposed: in the *q* = [*q*_0_, …, *q*_*J*_] list, *q* values are linearly sampled. A new list of *q* values, 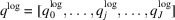 is created, in which values are logarithmically sampled between *q*_0_ and *q*_*J*_, and 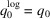 and 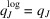 . The list of intensity values *I*_*n*_(*q* ^log^) is computed using linear interpolation from *I*_*n*_(*q*): *q* logarithmic scale (QLOG): *I*_*n*_(*q*) → *I*_*n*_(*q* ^log^).

Finally, to get rid of a problem of negative intensity due to the noise addition, the first preprocessing step will always be a thresholding operation at 10^−15^ cm^−1^, noted TH. The threshold value is defined empirically to always be lower than the signal.

As these preprocessing operations can be complementary and in order to optimize their action, they will be applied as composite functions. As an example, the notation TH ° LOG ° STD ° QLOG means that the TH function is applied on the *I*_*n*_ vector. The output of TH is then given as input to the LOG function, and so on up to the QLOG function. The output of the QLOG function will be used as input in the classification step. In this study, different preprocessing combinations were tried and all the results are available in Appendix *C* (see the supporting information).

Following the preprocessing, the second step of the classification method consists of projecting the input data in a latent space in which data will be compared by a classifier. As mentioned in the *Introduction*, we claim that the original space, *i.e.* the intensity space, is inefficient for the classification task because it does not allow us to capture the relevant pattern characteristics to differentiate efficiently the form factors. The transformation to the latent space has two objectives: firstly, it has to perform a dimensional reduction, which leads to a concentration of the information (Bengio *et al.*, 2013 ▸). In this study the intensity space has 890 dimensions (*i.e.* *J* = 889 in 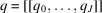 ). Secondly, it has to filter important information against useless information for the classification task. To define the transformation function, three types of methods are compared: transformation based on prior knowledge of SAXS theory, unsupervised representation learning and supervised representation learning. The classification on intensity space will be noted I and will be our baseline.

As the first space transformation, we propose to apply the transformation described by Franke *et al.* (2018 ▸), noted Franke_5_ in the following. For each simulation, the gyration radius *R*_g_ is estimated using the *AutoRg* program of *AtSAS* software (Petoukhov *et al.*, 2007 ▸). The new space is a six-features space, where the first features correspond to *V*′ quantities evaluated in five points and the last feature corresponds to *R*_g_. The transformation is defined as

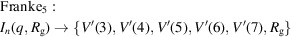

where 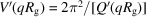 with

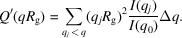

Due to the presence of form factors with inhomogeneous scattering length density in the data set, we decided to extend the upper *qR*_g_ bound to 7, in order to keep intraparticle contrast information present at higher *q*. The original transformation using the upper *qR*_g_ bound equal to 5 has also been tested on the data set with worse results; thus those results will not be presented in the following. We also tried a transformation with a less drastic dimension reduction: instead of computing *V*′ on five points, *V*′ is computed on 200 points linearly spaced in [3, 7]:

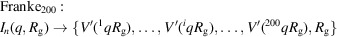

with ^*i*^ *qR*_g_ ∈ [3, 7].

It is important to notice that this method is not adapted to particles which have one dimension that exceeds the SAXS measurement range, as is the case for cylinders and core shell cylinders, which can be really long. Indeed, the notion of gyration radius loses its meaning for those form factors, and we will not apply these methods on them.

The second way to design the transformation is to use an unsupervised representation learning method to extract important information and to apply a dimension reduction. The first method of this type applied is principal components analysis (PCA) (Pearson, 1901 ▸), proposed by Tomaszewski *et al.* (2021 ▸), which allows us to summarize information in a smaller number of uncorrelated features. We apply PCA to our preprocessed data set in order to keep 90% of the variance. This choice leads us to keep the 37 first principal components. This transformation is noted PCA_90_.

The second unsupervised representation learning algorithm designed is a convolutional auto-encoder (CAE). It consists of two convolutional neural networks: an encoder which applies the dimension reduction from the intensity space to the latent space, and a decoder which takes the latent vector as input and reconstructs the intensity vector. The network is trained to output the intensity curve through the latent space. This forces the encoder to concentrate information in the latent space dimension, keeping maximum useful information to reconstruct the original input. The CAE is trained by backpropagation, the target being the same vector as the input, *i.e.* the preprocessed intensity curve. An Adam optimization is used with mean square error (MSE) as loss function. Different latent space dimensions have been tried: 10, 50 and 200. The training performs optimally with a 200 features latent space. The exact architecture is available in Appendix *D* (see the supporting information). All neural networks presented in the study have been trained on an Nvidia RTX 3080 GPU and have been implemented with *Keras* (v.2.4.3). A similar auto-encoder has been designed with fully connected layers instead of convolutional ones, but its performances were poor.

Finally, we propose to use supervised representation learning to design an optimal transformation. To do so, a convolutional neural network (CNN) is trained by backpropagation using preprocessed data as input and the corresponding form factor as target. This network is composed of two blocks: the convolutional block, referred to as CNN in the following, contains convolutional layers and max pooling operations and takes the preprocessed data as input and outputs a latent vector of dimension 256. The second block is a perceptron layer activated by a softmax function and used as classifier: it takes the latent vector as input and outputs a probability for each form factor. Once the network is trained, the CNN block can be used independently from the perceptron layer, in order to transform the data to the latent space. Nevertheless the perceptron layer can also be used as classifier, as described in the following section. The CNN block’s architecture has been optimized using neural architecture search methods (Jin *et al.*, 2019 ▸; Pham *et al.*, 2018 ▸) and is as follows:

1D convolutional layer [*n* filters: 64, kernel size: 7, activation function: ReLU (rectified linear unit)].

1D convolutional layer (*n* filters: 64, kernel size: 7, activation function: ReLU).

Max pooling operation (kernel size: 6).

Dropout operation (rate: 0.25).

1D convolutional layer (*n* filters: 64, kernel size: 7, activation function: ReLU).

1D convolutional layer (*n* filters: 256, kernel size: 7, activation function: ReLU).

Max pooling operation (kernel size: 6).

Dropout operation (rate: 0.25).

Global max pooling (output size: 256).

The network is trained using an Adam optimizer and categorical cross-entropy as loss function.

For each space transformation, the three classifiers that provided the best performance in the Tomaszewski *et al.* (2021 ▸) study have been compared. The first one is the KNN, which is a non-parametric classification method based on the Euclidean distance comparison between a test curve to be classified and training data set curves. Two classifiers based on decision trees are proposed: the random forest (RF) classifier (Verikas *et al.*, 2011 ▸), which consists of training independently several decision trees and making a decision based on a vote, and the extreme gradient boosting (XGBoost) classifier (Friedman, 2001 ▸), which consists of training each decision tree to correct the errors of the previously fitted ones. The last classifier presented is the perceptron layer activated with a softmax function. It is only employed in combination with the CNN transformation in our experiments, and while this is the most practical technique to train the network, it underperforms when compared with the other classifiers. KNN and RF are implemented using the *scikit-learn* Python package (v.0.24.1), XGBoost is implemented using the *XGBoost* Python package (v.1.6.0.) and the perceptron layer is implemented using *Keras* (v.2.4.3).

For each latent space, a hyper-parameter exploration is conducted for each classifier. As the computational costs necessary for the search of optimal hyper-parameters are very high, only limited sets of hyper-parameters have been tested. The list of hyper-parameters tested is available in Appendix *B* (see the supporting information). Once the hyper-parameters have been chosen, the different methods are tested by cross-validation on the whole data set divided using five folders.

**Figure 3:**
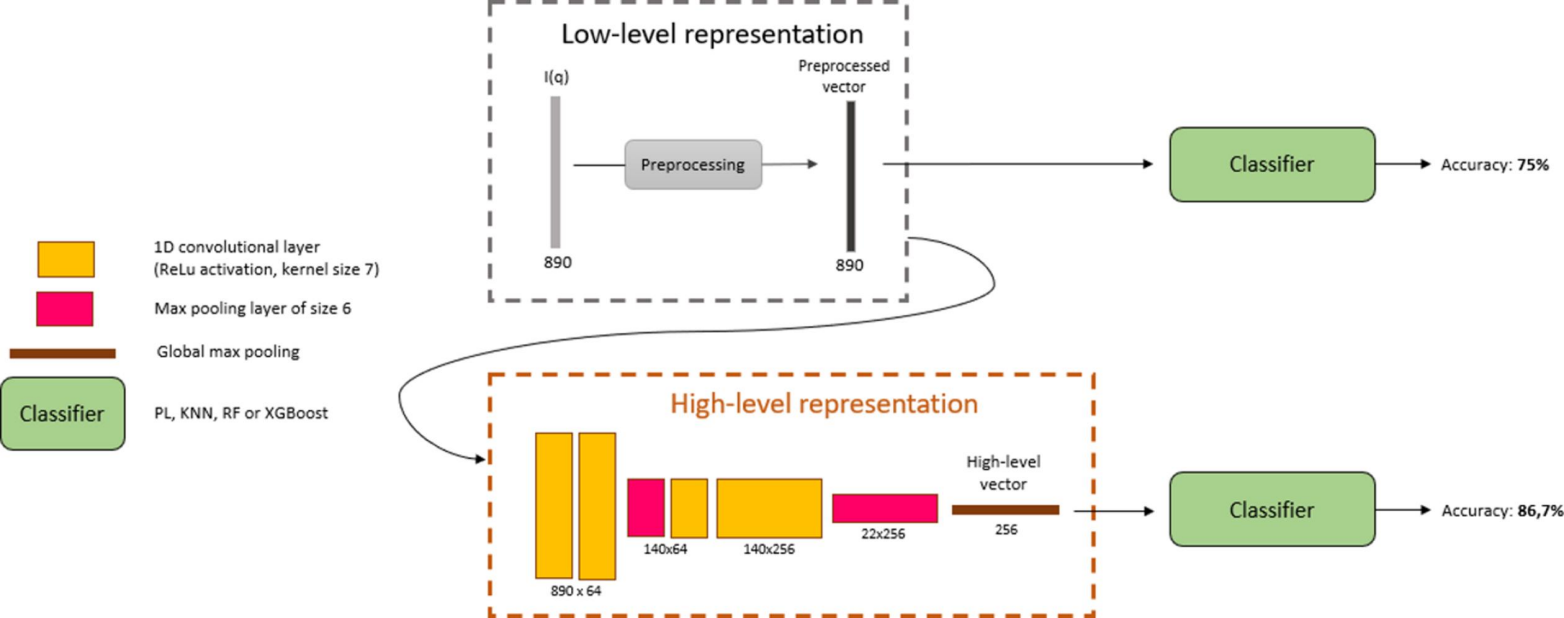
Representation of the classification in the intensity space versus in the CNN space.

Our approach is depicted in Fig. 3 ▸.

### 2.3. Influence of device configuration

SAXS devices are flexible machines with various parameters that can influence the data acquisition. Sample-to-detector distance, detector pixel size, beam size, beam center and acquisition time are a non-exhaustive list of the parameters that influence the measurement. Modification of these parameters leads to data with various *q*_min_, *q*_max_, sampling frequency δ*q* and more or less important smearing effects. In order to assess the transferability of the classification model between device configurations, the model has been trained on a data set with only Xeuss1800HR data, DS 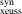 , or only NanoInXiderHR data, DS 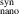 , and tested on synthetic data for both configurations. The classification model has also been trained on mixed data sets, mixing the two configurations. A data set, designed by DS 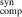 , contains all the synthetic samples, half in the NanoInXiderHR configuration and the other half in the Xeuss1800HR configuration. A data set designated by DS 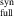 groups all the synthetic data in both configurations, and therefore contains twice as much data as the other data sets, but is based on the same number of noiseless data as the other data sets.

The two configurations lead to certain discrepancies in data: Xeuss1800HR data have a *q*_min_ = 0.85 × 10^−4^ Å^−1^, *q*_max_ = 1511 × 10^−4^ Å^−1^ and a sampling step Δ*q* = 1.7 × 10^−4^ Å^−1^. NanoInXiderHR data have a *q*_min_ = 3.74 × 10^−4^ Å^−1^, *q*_max_ = 4527 × 10^−4^ Å^−1^ and a sampling step Δ*q* = 7.5 × 10^−4^ Å^−1^. As NanoInXiderHR data are very noisy at high *q*, we have observed that setting *q*_max_ to 1511 × 10^−4^ Å^−1^ improves the prediction accuracy on this configuration. As the classification model requires a fixed input shape, NanoInXiderHR data are oversampled given the input shape using linear interpolation. The multi-configuration context requires another adaptation: when training and testing the model in a multi-configuration context, a *q*-values list is therefore a variable about which the model has no direct information if the input consists solely of the intensity values. To overcome this limitation, the model input is modified to accept a 2D input: (*I*, *q*).

### 2.4. Real data classification

To find out whether models trained on synthetic data can predict the form factors of real data, we produced data sets, DS 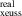 and DS 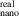 , from ten real samples, using two different devices. As the data set is very limited and does not include all the form factors, it is not possible to make a statistical evaluation of the model’s performance on those real data sets. Nevertheless, analysis of the model’s predictions can give us an idea of the transferability of the rules learned from synthetic data to real data. For the evaluation of predictions on real data, we propose to use a score incremented by +1 for a correct prediction, −1 for an erroneous prediction and 0 for an informative prediction. An informative prediction corresponds to a confusion with a similar form factor. For spheres, confusion with a prolate or oblate is considered informative. Similarly, for prolates, confusion with an oblate or sphere is informative. For the core shell sphere sample, confusion with a core shell prolate, core shell oblate or hollow sphere will be considered informative. A score of +10 or −10 corresponds to a situation where all the samples were, respectively, well classified and misclassified.

At small *q*, some samples show artifacts characteristic of large objects, certainly due to the onset of aggregation or to micro air bubbles suspended in the solution. Only the part of the curve with *q* > 0.005 Å^−1^ is retained for both synthetic training data and real test data. This leads to degraded performances on synthetic test data sets but it significantly improves results on real data.

## 3. Results and discussion

### 3.1. Model performances on synthetic data

For comparing the performances of the different approaches on the synthetic data set, accuracy is an appropriate measure since the class distribution is balanced throughout the data set. Furthermore, this metric is discriminant and easy to understand. The F1 score and Matthews correlation coefficient were also computed, but do not bring extra information. The main results of the representation space comparison are presented in Table 1 ▸. The best results according to a Wilcoxon rank sum test used at a *p*-value threshold of 0.01 (Lehmann & D’Abrera, 1975 ▸) are shown in bold. Different preprocessing combinations are evaluated for each latent space. In this section, just the combination that produces the best outcome will be detailed below; other tested combinations are supplied in Appendix *C* (see the supporting information). The training and testing operation is repeated 20 times, and the presented results are the average accuracy obtained over the 20 trials.

Applying the RF classifier directly on the preprocessed *I*(*q*) space allows us to reach 75.0% accuracy on the data set. The best method in terms of accuracy is the CNN ° XGBoost which performs with 86.7% accuracy. The perceptron layer (PL) does not appear in the table because it is used only with CNN, but the CNN ° PL performs with 83.4% accuracy.

PCA_90_ and CAE spaces lead to similar results for the different classifiers, and do not allow us to overcome the baseline performances. Overall, the performance of classification based on the CNN latent space far exceeds the performance of classification applied on other spaces. An explanation of this phenomenon is that the CNN space is the only space especially trained to be optimal for the targeted classification task. Unsupervised methods are trained to compress information without specifically selecting information useful for classification. It is interesting to note that for a given representation space, decision trees based methods have higher accuracy than KNN. This is interesting for prediction time: when a KNN-like algorithm’s computing cost is precisely proportional to the size of the database, the RF and XGBoost classifiers provide an instantaneous form factor prediction.

In both cases of CNN space and *I*(*q*) space, the preprocessing combination leading to the best performance is TH ° IntN ° LOG ° STD ° QLOG.

**Figure 4:**
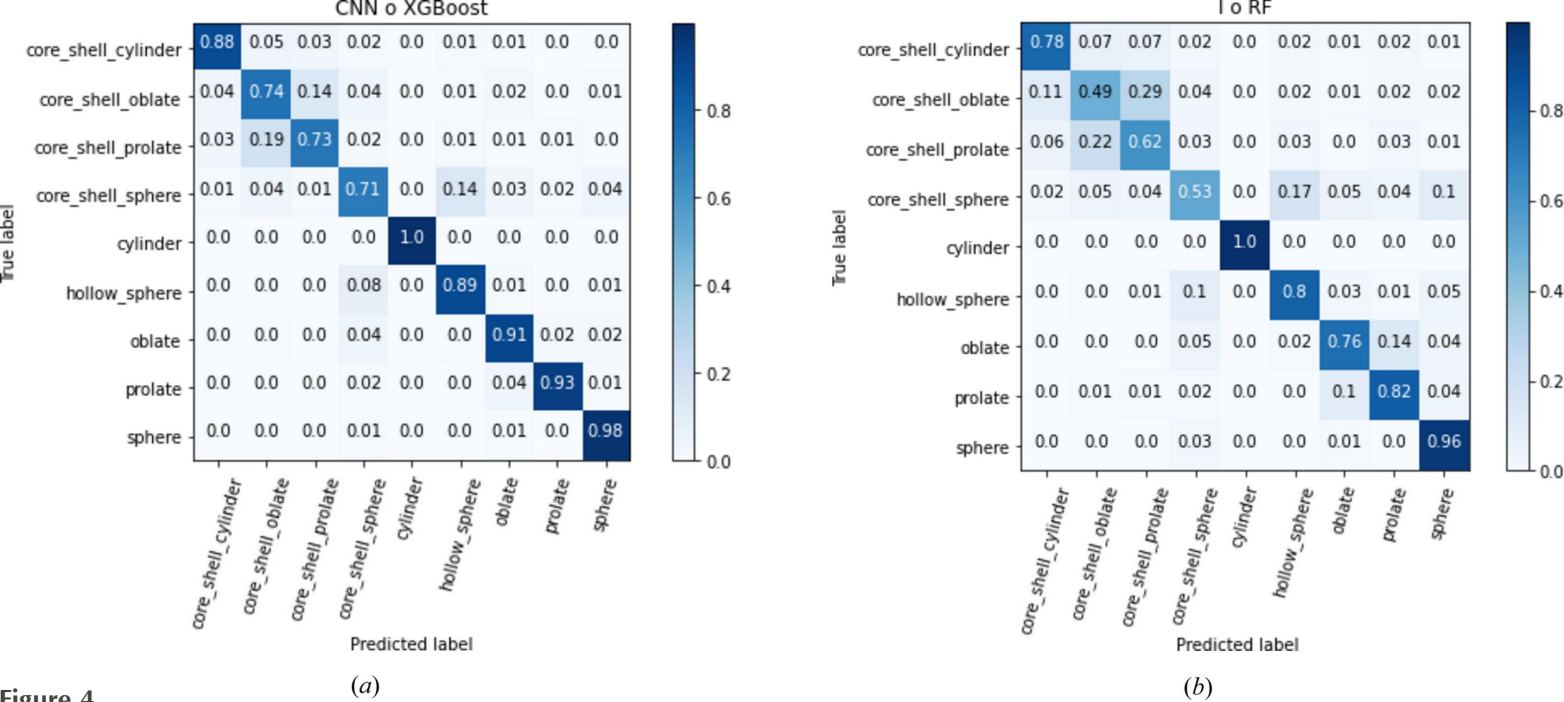
Confusion matrix of the (*a*) CNN ° XGBoost and (*b*) I ° RF methods on the whole data set. These confusion matrices are normalized by the number of predictions, so that the sum of a row is equal to 1. As an example, on (*a*) 93% of the prolates were well classified, 4% were confused with oblates, 2% with core shell spheres and 1% with spheres.

To understand the origin of the observed improvement of methods involving CNN space compared with methods involving I space, it is interesting to examine the details of performance by class in Fig. 4 ▸. We compare the confusion matrix (Ting, 2010 ▸) of the best I space classifier and of the best CNN space classifier, which are, respectively, the I ° RF method and the CNN ° XGBoost method. Regarding the confusion matrix of I ° RF in Fig. 4 ▸(*b*), the principal difficulties of this classification task lie in the classification of particles with inhomogeneous scattering length density. Indeed, homogeneous form factors have high accuracy: around 100% for cylinders and spheres, 76% for oblates and 82% for prolates. Most of the nonhomogeneous particles are predicted with a low accuracy: 49% for core shell oblates, 62% for core shell prolates and 53% for core shell spheres. Hollow spheres and core shell cylinders are better classified with 80% and 78%, respectively. A large part of the misclassified nonhomogeneous particles are confused with other nonhomogeneous particles. As an example, among the 38% of misclassified core shell prolates, 90% are confused with nonhomogeneous particles.

Let us now analyze the confusion matrix of the CNN ° XGBoost method in Fig. 4 ▸(*a*). The classification scores of homogeneous particles have been significantly improved with respect to I ° RF: prolate and oblate accuracy exceeds 90%. There is also significant improvement in nonhomogeneous particle classification: the ellipsoidal and spherical core shell particles rise from a 49–62% accuracy range to a 71–74% accuracy range. Nevertheless, those form factors still have a lot of misclassification. Analysis of the misclassification shows that, for a given nonhomogeneous form factor, the misclassified samples are largely confused with a unique other form factor, which is a more informative situation than when the confused form factor is random. The example of core shell prolate is representative: 70% of the misclassified core shell prolates are confused with core shell oblates. To summarize, training a convolutional neural network to project data from the intensity space into a space specifically adapted for the classification task allows the classifier to detect finer discriminant patterns in the data. It increases the prediction accuracy of every form factor that was not already maximal.

The classification on Franke’s space has been evaluated on a data set from which cylinders and core shell cylinders have been removed, so the average accuracy on this data set is not comparable with the results of Table 1 ▸. In order to have comparison points, baseline methods I ° RF were also evaluated on this data set. Classification on Franke’s space has performance below the baseline. Details of the Franke’s space results are available in Appendix *E* (see the supporting information).

**Figure 5:**
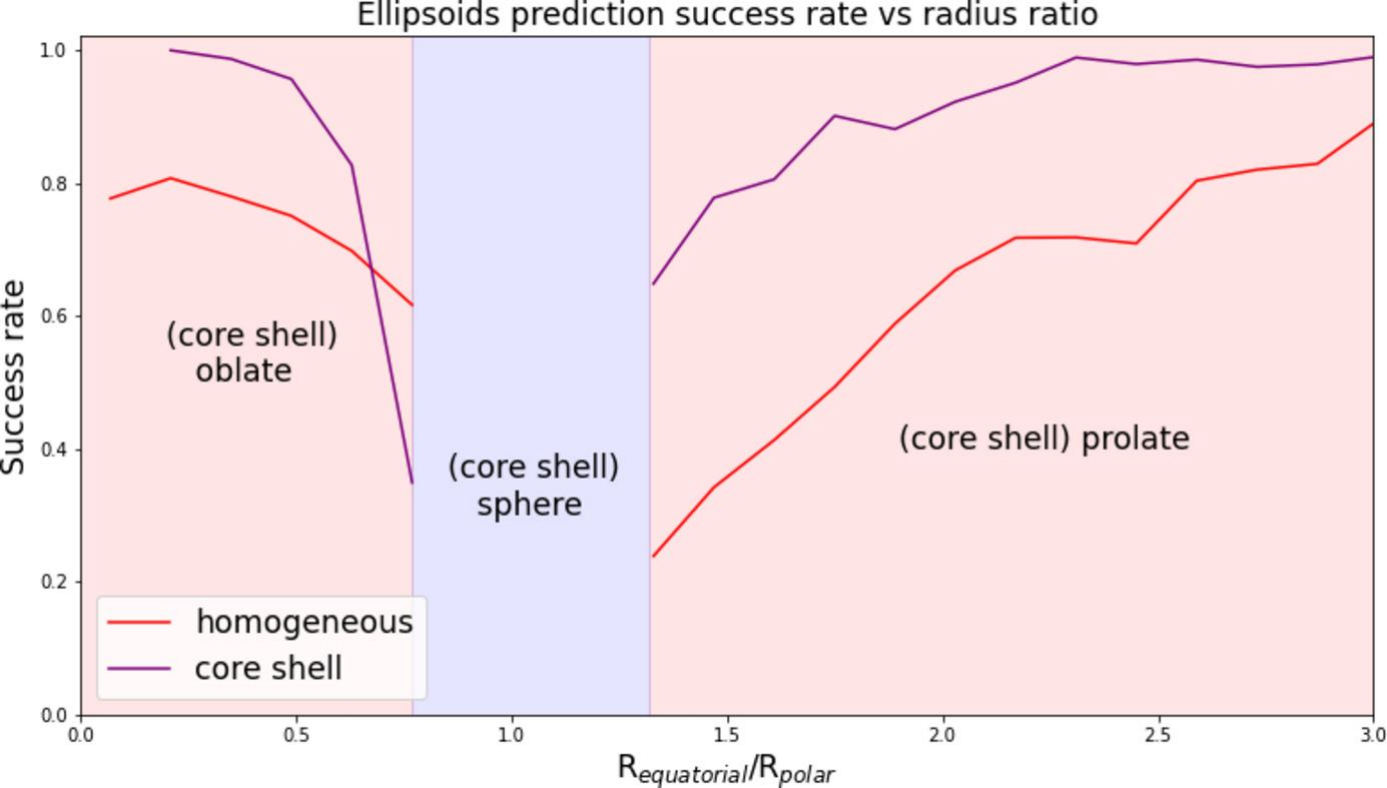
Prediction success rate when using CNN ° XGBoost for ellipsoid and core shell ellipsoid form factors versus their aspect ratio *R*_equatorial_/*R*_polar_. A ratio of 1 corresponds to a (core shell) spherical shape.

It is interesting to observe the influence of nanoparticle structural parameters on the predictor success rate. The influence of the aspect ratio for homogeneous and nonhomogeneous ellipsoids on CNN ° XGBoost predictor accuracy can be seen in Fig. 5 ▸. When the aspect ratio tends towards 1, *i.e.* when ellipsoids tend towards spherical shapes, we observe a significant decrease in prediction success rates, for both homogeneous and core shell ellipsoids. This example shows that the predictor’s performance is logically affected by the structural parameters of the nanoparticles, and that nanoparticles at the boundary between two form factors will generate high uncertainty in the prediction.

### 3.2. Multi-configuration training for model generalization

A sharp deterioration of prediction results is observed in Table 2 ▸(*a*) when a model trained on Xeuss1800HR data is tested on the NanoInXiderHR configuration. The same phenomenon is observed for a model trained on NanoInXiderHR data and tested on Xeuss1800HR data. It seems that the classification rules learned on a given device configuration are poorly transferable to another device configuration.

Training on mixed configuration data sets should enable the model to be generalized rather than specific to a single configuration. Training on DS 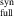 , which regroups all samples in both configurations, allows us to reach the accuracy level obtained on synthetic data for models trained specifically for a given configuration. We have further observed that adding the *q* vector as input to the model has improved accuracy by 2 to 4 points, both in the multi-configuration training context, which was expected, and in the single-configuration training, which is more surprising because the *q* vector does not vary during training in this context.

### 3.3. Transferability to real data classification

**Figure 6:**
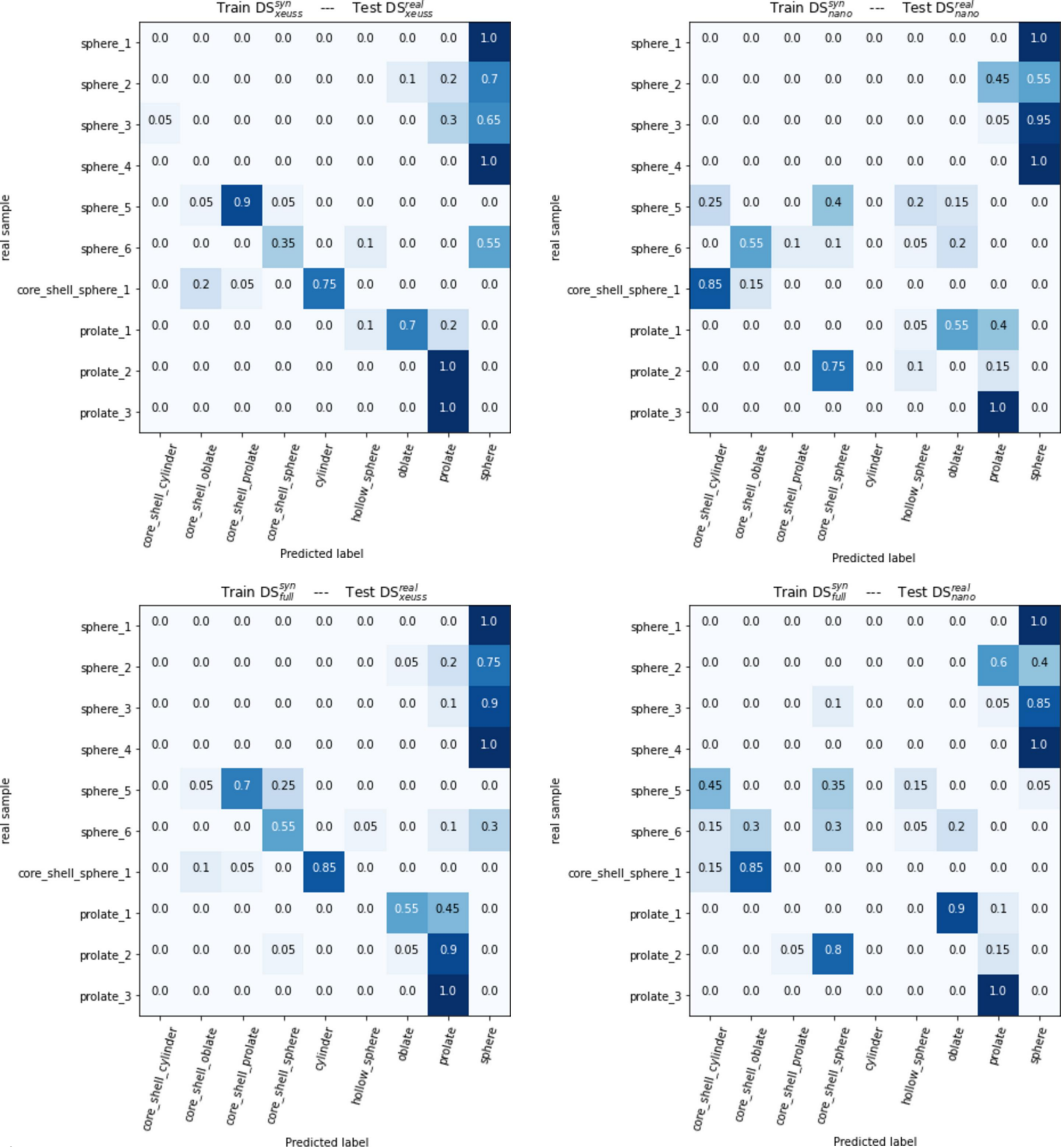
Details of predictions for each sample in DS 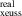 and DS 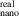 when the model is on DS 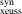 , DS 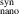 and DS 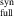 . Values correspond to the frequency with which a sample is classified as a given form factor over the 20 training sessions.

In this section we apply the classification model with best accuracy CNN ° XGBoost on the real data. The predictions are detailed for each measured sample in Fig. 6 ▸. On Figs. 6 ▸(*c*) and 6 ▸(*d*) we can see that training on DS 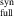 allows the model to make globally correct predictions for spheres No. 1 to No. 4, but fails to correctly classify spheres No. 5 and No. 6 for both configurations. The core shell sphere prediction is mostly confused with a cylinder in the Xeuss1800HR configuration, whereas in the NanoInXiderHR configuration it is classified as a core shell oblate, which seems to be close to reality according to TEM characterization [*cf.* Fig. 2 ▸(*b*)]. The three prolate ellipsoids are well classified overall with the Xeuss1800HR configuration, but the results with the NanoInXiderHR configuration exhibit diminished efficacy, since prolate No. 2 is often confused with a core shell sphere and prolate No. 1 is classified as an oblate.

Table 4 in Appendix *F*1 (see the supporting information) summarizes the fits and predictor results for the experimental data acquired on the Xeuss device. The samples that are always predicted correctly by the machine learning model (sphere No. 1, sphere No. 4, prolate No. 2 and prolate No. 3) correspond to very good quality SAXS curves which give an excellent quality fit with the expected form factor. The samples sphere No. 2, sphere No. 3 and sphere No. 6 are often confused by the predictor. For those three samples, the confused form factor leads to better fits than the expected form factor. Despite this, the predictor was still able to detect the correct form factor in the majority of training sessions. The samples sphere No. 5, core shell sphere No. 1, prolate No. 1 are almost always misclassified. In the first two cases, the expected and confused form factors lead to very poor quality fits, which may explain the difficulty of the predictor in proposing a consistent output. The prolate form factor fits the sample prolate No. 1 very well. However, the curve shows a residual pattern from buffer subtraction at low *q*, which could explain the predictor’s difficulty in choosing the right form factor, and suggests that the predictor is over-sensitive to this type of pattern.

It can be underlined that, for each sample, the predictions are very similar whether the model has been trained on the data-specific configuration or on DS 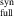 . This evaluation thus shows that a single model trained on several device configurations is competitive in terms of accuracy with a model dedicated to a single configuration, with a much improved versatility.

Predictions on some samples show that the predictor can produce more realistic results than the method of choosing the form factor by selecting the best fit, while eliminating parameter initialization and calculation time issues. Also, the model trained on synthetic data seems to be able to generalize the learned rules to real data. However, this generalization is far from perfect, because the predictor is over-sensitive to patterns intrinsic to real data that are not simulated in synthetic data. Thus, obtaining a more substantial experimental data set could be a way to fine-tune the model, as was done by Abdel Aty *et al.* (2022 ▸) in order to overcome this issue.

## 4. Conclusion

In this paper, we propose a supervised representation learning approach based on convolutional neural networks that automatically determines an optimized space transformation for SAXS data classification. We show that training several machine learning classifiers on the learned representation space improves their performance significantly on synthetic data when compared with training them on the original intensity space or on a transformed space found by other classical approaches. When training a classifier on the space generated by the suggested technique, our findings reveal that misclassification is nearly always caused by confusion with the most comparable form factor rather than confusion with different form factors when classification is done in intensity space.

We show that classification rules learned on a single device configuration transfer poorly to other device configurations, from one device configuration to another, while demonstrating that a model trained on several configurations performs as well as a model dedicated to one configuration, which reduces the number of models needed to create an efficient classification applicable to multiple experimental conditions. Using a small experimental data set of real samples on several laboratory devices allowed us to show that classification rules learned on real data are partially transferable to real data classification. The methodology presented here has the potential for creating an easy-to-use classification application, which could enable the user to quickly select the optimal form factor to use for fitting the data with the appropriate deterministic SAXS model, or which could be applied to real-time quality control use-cases as well as cases involving the management of large quantities of data. To achieve this, several directions will be explored in future work. To evaluate the robustness of the machine learning model, validation on a more complete real data set is required. Access to such a data set will also enable us to improve performance on real data by using few shot learning. Also, a more in-depth assessment of robustness to noise and variety of configurations, and the implementation of an out-of-distribution data detector are essential to avoid major confusion and to give users confidence in the predictions. Finally, using machine learning to speed up the fitting process could also lead to a complete and versatile nanoparticle analysis application.

## Supplementary Material

Appendices A to F. DOI: 10.1107/S2053273324000950/iv5034sup1.pdf

The dataset used to train and test the algorithms: https://dx.doi.org/10.17632/b96sw3jffy.2

## Figures and Tables

**Table 1:** % accuracy of different classifiers applied on different data representations with the best preprocessing combination Uncertainty corresponds to 3σ, with σ the standard deviation of the accuracy over the different training sessions. For each classifier, results in bold are significantly the best with respect to a Wilcoxon rank test at a *p*-value threshold of 0.01 (Lehmann & D’Abrera, 1975 ▸).

Representation	KNN	RF	XGBoost
*I*(*q*) space	55.4 ± 0.0	75.0 ± 0.9	73.8 ± 1.1
PCA_90_	64.9 ± 0.3	69.5 ± 1.6	70.0 ± 1.8
CAE	66.5 ± 1.3	69.6 ± 1.0	70.1 ± 1.5
CNN	**84.1 ± 1.1**	**84.9 ± 1.2**	**86.7 ± 1.6**

**Table:** 

	Test DS	
Train DS	DS 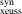	DS 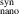
(*a*) Accuracy (%) for synthetic test data sets in single- and multi-configuration training contexts. Uncertainty corresponds to 3σ, with σ the standard deviation of the metric over the different training sessions		
DS 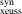	88.5 ± 0.8	44.5 ± 4.8
DS 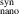	56.1 ± 4.2	86.8 ± 1.2
DS 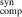	85.2 ± 1.3	85.2 ± 1.7
DS 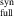	87.5 ± 1.1	87.4 ± 0.8

**Table:** 

	Test DS	
Train DS	DS 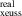	DS 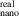
(*b*) Score for real test data sets in single- and multi-configuration training contexts. Uncertainty on score corresponds to 3σ, with σ the standard deviation of scores over the different training sessions		
DS 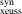	4.35 ± 0.75	−4.65 ± 0.70
DS 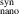	1.85 ± 0.72	1.60 ± 0.79
DS 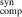	3.80 ± 0.77	2.25 ± 0.81
DS 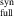	4.40 ± 0.77	1.90 ± 0.81

## References

[bb1] Abdel Aty, H., Strutt, R., Mcintyre, N., Allen, M., Barlow, N. E., Páez-Pérez, M., Seddon, J. M., Brooks, N., Ces, O. & Gould, I. R. (2022). *Digital Discovery*, **1**, 98–107.

[bb2] Allec, N., Choi, M., Yesupriya, N., Szychowski, B., White, M. R., Kann, M. G., Garcin, E. D., Daniel, M.-C. & Badano, A. (2015). *Sci. Rep.* **5**, 12085. 10.1038/srep12085 PMC4498188 26160052

[bb3] Archibald, R. K., Doucet, M., Johnston, T., Young, S. R., Yang, E. & Heller, W. T. (2020). *J. Appl. Cryst.* **53**, 326–334.

[bb4] Astudillo, R. F., Amir, S., Ling, W., Silva, M. J. & Trancoso, I. (2015). *Proceedings of the 53rd Annual Meeting of the Association for Computational Linguistics and the 7th International Joint Conference on Natural Language Processing* (Volume 1: Long Papers), pp. 1074–1084.

[bb5] Bakry, M., Haddar, H. & Bunău, O. (2019). *J. Appl. Cryst.* **52**, 926–936.

[bb6] Bengio, Y., Courville, A. & Vincent, P. (2013). *IEEE Trans. Pattern Anal. Mach. Intell.* **35**, 1798–1828. 10.1109/TPAMI.2013.50 23787338

[bb7] Benvenuto, F., Haddar, H. & Lantz, B. (2016). *SIAM J. Appl. Math.* **76**, 276–292.

[bb8] Boldon, L., Laliberte, F. & Liu, L. (2015). *Nano Rev.* **6**, 25661. 10.3402/nano.v6.25661 PMC4342503 25721341

[bb9] Boulanger-Lewandowski, N., Yoshua, B. & Pascal, V. (2012). arXiv:1206.6392.

[bb10] Da Vela, S. & Svergun, D. I. (2020). *Curr. Res. Struct. Biol.* **2**, 164–170. 10.1016/j.crstbi.2020.08.004 PMC8244429 34235476

[bb11] De Geuser, F. & Deschamps, A. (2012). *C. R. Phys.* **13**, 246–256.

[bb12] Do, C., Chen, W.-R. & Lee, S. (2020). *MRS Adv.* **5**, 1577–1584.

[bb13] Fong, A. Y., Pellouchoud, L., Davidson, M., Walroth, R. C., Church, C., Tcareva, E., Wu, L., Peterson, K., Meredig, B. & Tassone, C. J. (2021). *J. Chem. Phys.* **154**, 224201. 10.1063/5.0047385 34241189

[bb14] Franke, D., Jeffries, C. M. & Svergun, D. I. (2018). *Biophys. J.* **114**, 2485–2492. 10.1016/j.bpj.2018.04.018 PMC6129182 29874600

[bb15] Friedman, J. H. (2001). *Ann. Statist.* pp. 1189–1232.

[bb16] Guinier, A., Fournet, G. & Yudowitch, K. L. (1955). *Small-Angle Scattering of X-rays*, pp. 156–160. New York: Wiley.

[bb17] Hinton, G., Deng, L., Yu, D., Dahl, G. E., Mohamed, A., Jaitly, N., Senior, A., Vanhoucke, V., Nguyen, P., Sainath, T. & Kingsbury, B. (2012). *IEEE Signal Process. Mag.* **29**, 82–97.

[bb18] Huang, J., Li, Y.-F. & Xie, M. (2015). *Inf. Softw. Technol.* **67**, 108–127.

[bb19] Jin, H., Song, Q. & Hu, X. (2019). *Proceedings of the 25th ACM SIGKDD International Conference on Knowledge Discovery and Data Mining*, pp. 1946–1956. ACM, Association for Computing Machinery.

[bb20] Lehmann, E. L. & D’Abrera, H. J. (1975). *Nonparametrics: Statistical Methods Based on Ranks.* Holden-Day.

[bb21] Li, T., Senesi, A. J. & Lee, B. (2016). *Chem. Rev.* **116**, 11128–11180. 10.1021/acs.chemrev.5b00690 27054962

[bb22] Liu, S., Melton, C. N., Venkatakrishnan, S., Pandolfi, R. J., Freychet, G., Kumar, D., Tang, H., Hexemer, A. & Ushizima, D. M. (2019). *MRS Commun.* **9**, 586–592.

[bb23] Miranda, S. M., Romanos, G. E., Likodimos, V., Marques, R. R. N., Favvas, E. P., Katsaros, F. K., Stefanopoulos, K. L., Vilar, V. J. P., Faria, J. L., Falaras, P. & Silva, A. M. T. (2014). *Appl. Catal. Environ.* **147**, 65–81.

[bb24] Molodenskiy, D. S., Svergun, D. I. & Kikhney, A. G. (2022). *Structure*, **30**, 900–908. 10.1016/j.str.2022.03.011 35413244

[bb25] Monge, N. (2023). *SAXS Nanoparticles for Machine Learning.* https://doi.org/10.17632/b96sw3jffy.1.

[bb26] Moon, T. K. (1996). *IEEE Signal Process. Mag.* **13**, 47–60.

[bb27] Pearson, K. (1901). *London, Edinb. Dubl. Philos. Mag. J. Sci.* **2**, 559–572.

[bb28] Petoukhov, M. V., Konarev, P. V., Kikhney, A. G. & Svergun, D. I. (2007). *J. Appl. Cryst.* **40**, 223–228.

[bb29] Pham, H., Guan, M., Zoph, B., Le, Q. & Dean, J. (2018). *International Conference on Machine Learning*, pp. 4095–4104. PMLR, Proceedings of Machine Learning Research.

[bb30] Portale, G., Cavallo, D., Alfonso, G. C., Hermida-Merino, D., van Drongelen, M., Balzano, L., Peters, G. W. M., Goossens, J. G. P. & Bras, W. (2013). *J. Appl. Cryst.* **46**, 1681–1689.

[bb31] Talapin, D. V. & Shevchenko, E. V. (2016). *Chem. Rev.* **116**, 10343–10345. 10.1021/acs.chemrev.6b00566 27677520

[bb32] Ting, K. M. (2010). *Encyclopedia of Machine Learning*, edited by C. Sammut & G. I. Webb, p. 209. Springer.

[bb33] Tobler, D. J., Shaw, S. & Benning, L. G. (2009). *Geochim. Cosmochim. Acta*, **73**, 5377–5393.

[bb34] Tomaszewski, P., Yu, S., Borg, M. & Rönnols, J. (2021). *Mach. Learn.* pp. 1–6.

[bb35] Tung, C. H., Chang, S. Y., Chen, H. L., Wang, Y., Hong, K., Carrillo, J. M., Sumpter, B. G., Shinohara, Y., Do, C. & Chen, W. R. (2022). *J. Chem. Phys.* **156**, 131101. 10.1063/5.0086311 35395880

[bb36] Veit, A., Alldrin, N., Chechik, G., Krasin, I., Gupta, A. & Belongie, S. (2017). *Proceedings of the IEEE Conference on Computer Vision and Pattern Recognition*, pp. 839–847.

[bb37] Verikas, A., Gelzinis, A. & Bacauskiene, M. (2011). *Pattern Recognit.* **44**, 330–349.

