# Supplementary material for: Automated selection of nanoparticle models for small-angle X-ray scattering data analysis using machine learning
Source: Acta Crystallogr A Found Adv. 2024 Feb 29;80(Pt 2):202–12. doi: 10.1107/S2053273324000950 (PMC10913671; doi:10.1107/S2053273324000950)
Supplement: Supplementary file 1 [file a-80-00202-sup1.pdf]

FOUNDATIONS
ADVANCES

**Volume 80 (2024)**

**Supporting information for article:**

# Automated selection of nanoparticle models for small-angle X-ray scattering data analysis using machine learning

**Nicolas Monge, Alexis Deschamps and Massih-Reza Amini**

# Supporting information for paper:
Automated selection of nanoparticle models for SAXS data analysis using machine learning

Nicolas MONGE[1,2,3], Alexis DESCHAMPS[2], and Massih-Reza AMINI[3]

[1]Xenocs, Grenoble, France
[2]SIMaP, University Grenoble Alpes, CNRS, Grenoble INP, Grenoble, France
[3]LIG, University Grenoble Alpes, CNRS, Grenoble, France

## Glossary

**$PCA_{90}$** PCA where 90% of the variance is conserved. 6

## Acronyms

**CAE** Convolutional Auto Encoder. 6, 8

**CNN** Convolutional Neural Network. 6

**KNN** K-Nearest Neighbors. 6

**RF** Random Forest. 6

**TEM** Transmission Electron Microscopy. 12

**XGBoost** eXtreme Gradient Boosting. 6

# A Data generation

## A.1 Simulation parameters

This section contains all information about the data set used in this study. The distribution of form factors in the database is balanced with 4.184 $I(q)$ curves simulated per form factor, which improves the interpretation of the results. As a result, the density of the parameter space varies according to the number of shape factor parameters, but this makes it possible to retain a significant number of simulations for form factors with few parameters. For the 9 form factor used, the following list details how parameters has been chosen. For each occurrence of form factor simulation, variable parameters are drafted following a uniform law. For some parameter, restrictions are added. When mentioned, the parameter is poly-dispersed. The poly-dispersion function is a Gaussian with a full width at half maximum equal to $a \times param$ with $a$ randomly selected following a uniform law on [0, 0.3].

Variable parameters

- Sphere:
  - Radius: $[50, 1000]\mathring{A}$, log-distribution, poly-dispersed
  - Scattering length density: $[5, 131] \times 10^6 \mathring{A}^{-2}$, linear distribution

  (https://www.sasview.org/docs/user/models/sphere.html)
- Oblate:
  - Radius equat:$[50, 1000]\mathring{A}$, log-distribution, poly-dispersed
  - Coeff Radius polar: $[0.1, 0.77]$, linear distribution, poly-dispersed
  - Scattering length density: $[5, 131] \times 10^6 \mathring{A}^{-2}$, linear distribution

  (https://www.sasview.org/docs/user/models/ellipsoid.html)
- Prolate:
  - Radius equat:$[50, 1000]\mathring{A}$, log-distribution, poly-dispersed
  - Coeff Radius polar: $[1.3, 5]$, linear distribution, poly-dispersed
  - Scattering length density: $[5, 131] \times 10^6 \mathring{A}^{-2}$, linear distribution

  (https://www.sasview.org/docs/user/models/ellipsoid.html)
- Cylinder:
  - Radius: $[50, 1000]\mathring{A}$, log-distribution, poly-dispersed
  - Length: $[100, 200] \times radius$, linear distribution, poly-dispersed
  - Scattering length density: $[5, 131] \times 10^6 \mathring{A}^{-2}$ linear distribution

  (https://www.sasview.org/docs/user/models/cylinder.html)
- Core Shell Sphere:
  - Radius core: $[50, 950]\mathring{A}$, log-distribution, poly-dispersed
  - Shell thickness: $[50, 950]\mathring{A}$, log-distribution, poly-dispersed
  - radius core + shell thickness $\leqslant 1000\mathring{A}$
  - Scattering length density core: $[5, 131] \times 10^6 \mathring{A}^{-2}$, linear distribution
  - Scattering length density shell: $[5, 131] \backslash [0.9 sld_{core}; 1.1 sld_{core}] \times 10^6 \mathring{A}^{-2}$, linear distribution

  (https://www.sasview.org/docs/user/models/core_shell_sphere.html)
- Hollow Sphere:
  - Radius core: $[50, 950]\mathring{A}$, log-distribution, poly-dispersed
  - Shell thickness: $[50, 950]\mathring{A}$, log-distribution, poly-dispersed

- radius core + shell thickness $\leqslant 1000\AA$
- Scattering length density core: same as solvent scattering length density
- Scattering length density shell: $[5, 131] \backslash [0.9 sld_{core}; 1.1 sld_{core}] \times 10^6 \AA^{-2}$, linear distribution

(https://www.sasview.org/docs/user/models/core_shell_sphere.html)

- Core Shell Oblate:
  - Radius equat: $[50, 950]\AA$, log-distribution, poly-dispersed
  - Coeff Radius polar: $[0.1, 0.77]$, linear distribution, linear distribution, poly-dispersed
  - Shell thickness: $[50, 950]\AA$, log-distribution, poly-dispersed
  - radius equat + shell thickness $\leqslant 1000\AA$
  - Scattering length density core: $[5, 131] \times 10^6 \AA^{-2}$, linear distribution
  - Scattering length density shell: $[5, 131] \backslash [0.9 sld_{core}; 1.1 sld_{core}] \times 10^6 \AA^{-2}$, linear distribution

(https://www.sasview.org/docs/user/models/core_shell_ellipsoid.html)

- Core Shell Prolate:
  - Radius equat: $[50, 950]\AA$, log-distribution, poly-dispersed
  - Coeff Radius polar: $[1.3, 5]$, linear distribution, linear distribution, poly-dispersed
  - Shell thickness: $[50, 950]\AA$, log-distribution, poly-dispersed
  - radius equat + shell thickness $\leqslant 1000\AA$
  - Scattering length density core: $[5, 131] \times 10^6 \AA^{-2}$, linear distribution
  - Scattering length density shell: $[5, 131] \backslash [0.9 sld_{core}; 1.1 sld_{core}] \times 10^6 \AA^{-2}$, linear distribution

(https://www.sasview.org/docs/user/models/core_shell_ellipsoid.html)

- Core Shell Cylinder:
  - Radius: $[50, 1000]\AA$, log-distribution, poly-dispersed
  - Length: $[100, 200] \times radius$, log-distribution, poly-dispersed
  - Shell thickness: $[50, 1000]\AA$, log-distribution, poly-dispersed
  - radius + shell thickness $\leqslant 1000\AA$
  - Scattering length density core: $[5, 131] \times 10^6 \AA^{-2}$, linear distribution
  - Scattering length density shell: $[5, 131] \backslash [0.9 sld_{core}; 1.1 sld_{core}] \times 10^6 \AA^{-2}$, linear distribution

(https://www.sasview.org/docs/user/models/core_shell_cylinder.html)

## A.2 Curves examples' parameters

The parameters used to simulate the curves shown in Figure 1 are as follows:

- Core shell cylinder
  - Radius: $100\AA$
  - Length: $5000\AA$
  - Shell thickness: $100\AA$
  - Scattering length density core: $20 \times 10^6 \AA^{-2}$
  - Scattering length density shell: $10 \times 10^6 \AA^{-2}$
- Core Shell Prolate:
  - Radius equat: $100\AA$

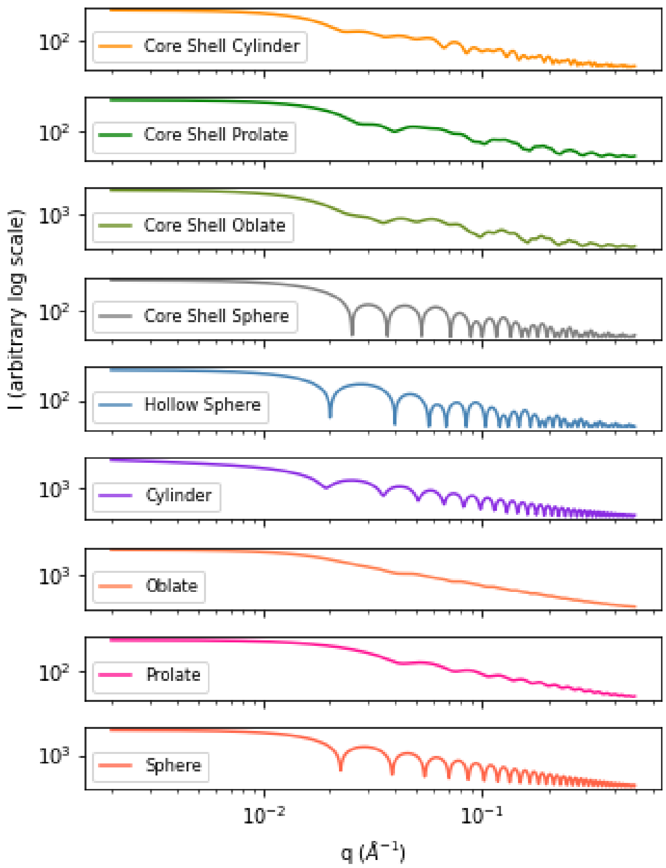


Figure 1: Example of noiseless I(q) curves generated using the 9 form factors, all particle sizes having the same order of magnitude and all particles having the same scattering length density.

  - Radius polar: 50$\AA$
  - Shell thickness: 100$\AA$
  - Scattering length density core: $20 \times 10^6 \AA^{-2}$
  - Scattering length density shell: $10 \times 10^6 \AA^{-2}$

- Core Shell Oblate:
  - Radius equat: 100$\AA$
  - Radius polar: 200$\AA$
  - Shell thickness: 100$\AA$
  - Scattering length density core: $20 \times 10^6 \AA^{-2}$
  - Scattering length density shell: $10 \times 10^6 \AA^{-2}$

- Core Shell Sphere:
  - Radius: 100$\AA$
  - Shell thickness: 100$\AA$
  - Scattering length density core: $20 \times 10^6 \AA^{-2}$
  - Scattering length density shell: $10 \times 10^6 \AA^{-2}$

- Hollow Sphere:

- Radius: $100\mathring{A}$
- Shell thickness: $100\mathring{A}$
- Scattering length density core: $0 \times 10^6 \mathring{A}^{-2}$
- Scattering length density shell: $10 \times 10^6 \mathring{A}^{-2}$

- Cylinder:
  - Radius: $200\mathring{A}$
  - Length: $5000\mathring{A}$
  - Scattering length density: $20 \times 10^6 \mathring{A}^{-2}$

- Oblate:
  - Radius equat:$200\mathring{A}$
  - Radius polar: $100\mathring{A}$
  - Scattering length density: $20 \times 10^6 \mathring{A}^{-2}$

- Prolate:
  - Radius equat:$100\mathring{A}$
  - Radius polar: $200\mathring{A}$
  - Scattering length density: $20 \times 10^6 \mathring{A}^{-2}$

- Sphere:
  - Radius: $200\mathring{A}$
  - Scattering length density: $20 \times 10^6 \mathring{A}^{-2}$

There is no polydispersity in parameters of those examples.

# B Classifiers hyper-parameters

For each classifier, several hyper-parameters were tested. Other hyper-parameter are the default parameters of the scikit-learn package.

K-Nearest Neighbors (KNN) tested hyper-parameters:

- number of neighbors: 1, 3, 5, 7, 11, 21
- weights: distance, uniform

KNN best set of hyper-parameters founds:

- I $\circ$ KNN: number of neighbors=1, weights=uniform
- $PCA_{90}$ $\circ$ KNN: number of neighbors=3, weights=distance
- CAE $\circ$ KNN: number of neighbors=5, weights=distance
- CNN $\circ$ KNN: number of neighbors=11, weights=distance

Random Forest (RF) tested hyper-parameters:

- number of trees: 10, 40, 100, 200

RF best set of hyper-parameters founds:

- I $\circ$ RF: number of trees: 200
- $PCA_{90}$ $\circ$ RF: number of trees: 200
- CAE $\circ$ RF: number of trees: 200
- CNN $\circ$ RF: number of trees: 200

eXtreme Gradient Boosting (XGBoost) tested hyper-parameters:

- number of estimators: 10, 40, 100, 200

Other hyper-parameters are the default one in the
XGBoost best set of hyper-parameters founds:

- I $\circ$ XGBoost: number of trees: 200
- $PCA_{90}$ $\circ$ XGBoost: number of trees: 200
- CAE $\circ$ XGBoost: number of trees: 200
- CNN $\circ$ XGBoost: number of trees: 200

# C Preprocessing selection

Several combinations of preprocessings were tried for each representation space:

- $TH \circ LOG$
- $TH \circ I0 \circ LOG$
- $TH \circ LOG \circ STD$
- $TH \circ I0 \circ LOG \circ QLOG$
- $TH \circ IntN \circ LOG \circ STD$
- $TH \circ I0 \circ LOG \circ STD \circ QLOG$
- $TH \circ IntN \circ LOG \circ STD \circ QLOG$
- $TH \circ IntN \circ STD$
- $TH \circ IntN \circ STD \circ QLOG$

# D Convolutional Auto-Encoder architecture

There is the architecture of the Convolutional Auto Encoder (CAE):

Encoder:

- 1D convolutional layer (n filters: 64, kernel size: 7, activation function: ReLu)
- 1D convolutional layer (n filters: 64, kernel size: 7, activation function: ReLu)
- Max Pooling operation (kernel size: 6)
- Flatten layer
- Fully connected layer (n filters: 16, activation function: ReLu)
- Fully connected layer (n filters: latent dimension, activation function: linear)

Decoder:

- Fully connected layer (n filters: 148×64, activation function: ReLu)
- Reshape layer (output shape: (148, 64))
- UpSampling1D layer (upsampling factor: 6)
- ZeroPadding1D layer (padding: 1)
- 1D convolutional layer (n filters: 64, kernel size: 7, activation function: ReLu)
- 1D convolutional layer (n filters: 1, kernel size: 7, activation function: ReLu)

# E Results using Franke's space

Table 1 summarizes the main results obtained using Franke space on $DS^{syn}_{xeuss}$ from which the cylinders and core shell cylinders have been removed.

Table 1: Accuracy computed by cross-validation on the data set from which cylinders and core shell cylinders have been removed

| | Accuracy of classifiers (%) | | |
|---|---|---|---|
| Representation space | KNN | RF | XGBoost |
| I(q) space | 47.6 | 72.6 | 71.1 |
| $Franke_5$ | 38.7 | 65.9 | 67.4 |
| $Franke_{200}$ | 44.3 | 69.0 | 68.3 |

# F Experimental data

## F.1 Fits of experimental data

To better understand the predictor's predictions on the experimental data, it is interesting to evaluate the quality of the fits that can be made with the predicted form factors. We performed fits for each of the experimental curves obtained from the Xeuss, using the form factors most frequently predicted by classification models trained on $DS^{syn}_{xeuss}$. The fits are represented in appendix F.2 and their obtained $\chi^2$ are as follows:

- Sphere n°1:
  - Fit sphere: $\chi^2 = 2.68$
- Sphere n°2:
  - Fit sphere: $\chi^2 = 2.36$
  - Fit prolate: $\chi^2 = 1.13$
- Sphere n°3:
  - Fit sphere: $\chi^2 = 6.91$
  - Fit prolate: $\chi^2 = 5.21$
- Sphere n°4:
  - Fit sphere: $\chi^2 = 1.20$
- Sphere n°5: a residual pattern from buffer substraction appear at low q. A sphere and core shell sphere form factor has been used to fit the whole curve, and another fit with sphere form factor has been realized without the beginning of the curve.
  - Fit sphere whole curve: $\chi^2 = 260$
  - Fit core shell sphere whole curve: $\chi^2 = 212$
  - Fit sphere for $q > 0.003\mathring{A}^{-1}$: $\chi^2 = 3.11$
- Sphere n°6:
  - Fit sphere: $\chi^2 = 18.5$
  - Fit core shell sphere: $\chi^2 = 9.92$
- Core shell sphere n°1:
  - Fit core shell sphere: $\chi^2 = 154$
  - Fit sphere: $\chi^2 = 335$
  - Fit cylinder: $\chi^2 = 1190$
- Prolate n°1: a residual pattern from buffer substraction appear at low q.
  - Fit prolate: $\chi^2 = 1.53$
  - Fit oblate: $\chi^2 = 3.90$
- Prolate n°2:
  - Fit prolate: $\chi^2 = 1.12$
- Prolate n°3:
  - Fit prolate: $\chi^2 = 1.08$

Table 2: Results from predictors and fits and quality of experimental data

| Sample | Accurate prediction frequency | Best form factor to fit | Best fit quality | SAXS curve quality |
|---|---|---|---|---|
| Sphere n°1 | 20/20 | sphere | Excellent | Excellent |
| Sphere n°2 | 14/20 | prolate | Excellent | Excellent |
| Sphere n°3 | 13/20 | prolate | Medium | Excellent |
| Sphere n°4 | 20/20 | sphere | Excellent | Excellent |
| Sphere n°5 | 0/20 | core shell sphere | Bad | Bad |
| Sphere n°6 | 11/20 | core shell sphere | Medium | Medium |
| Core shell sphere n°1 | 0/20 | core shell sphere | Bad | Medium |
| Prolate n°1 | 4/20 | prolate | Excellent | Medium |
| Prolate n°2 | 20/20 | prolate | Excellent | Excellent |
| Prolate n°3 | 20/20 | prolate | Excellent | Excellent |

## F.2 TEM images and SAXS curves

Figures 2, 3, 4, 5, 6, 7, 8, 9, 10, 11 present a Transmission Electron Microscopy image of each real sample, corresponding SAXS curve in both device configuration and fits of Xeuss1800HR SAXS curves with various form factors. Some experimental aspect ratios have been measured using the TEM images: particles in the core shell sphere n°1 sample have an average aspect ratio of 1.16 between equatorial radius and polar radius, and then are between our definition of core shell sphere and core shell prolate, so we decided to label them as core shell sphere. In samples sphere n°2 and sphere n°5 the average aspect ratio is 1.10 and these samples are then labelled as sphere. For sample sphere n°1, the average aspect ratio is 1.01 and it is then labelled as sphere.

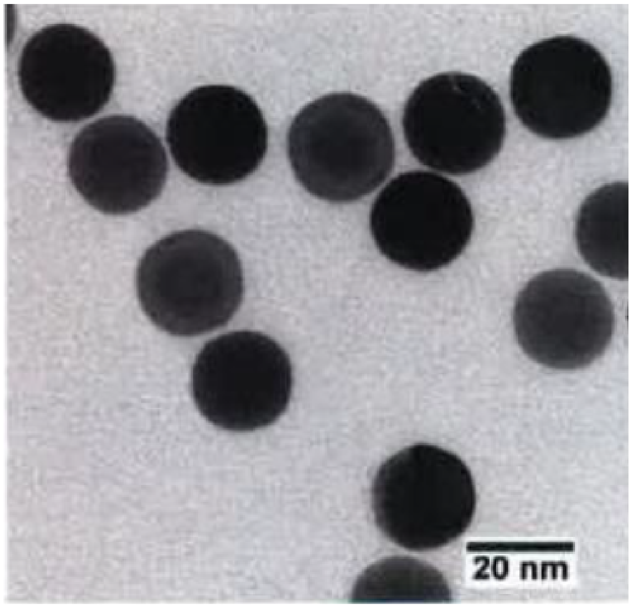


(a) TEM image of sphere n°1

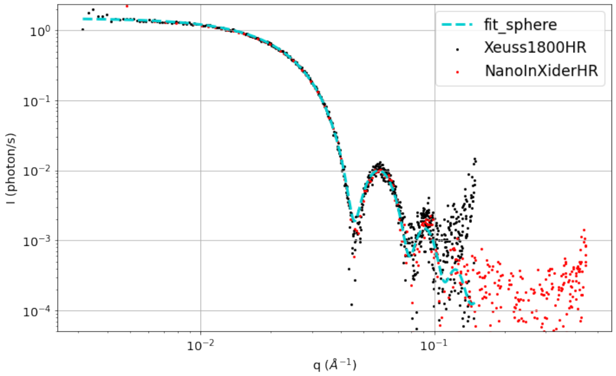


(b) SAXS curves of sphere n°1

Figure 2: TEM imaging, SAXS curve recorded on Xenocs devices and fit of the Xeuss1800HR curve for sample sphere n°1

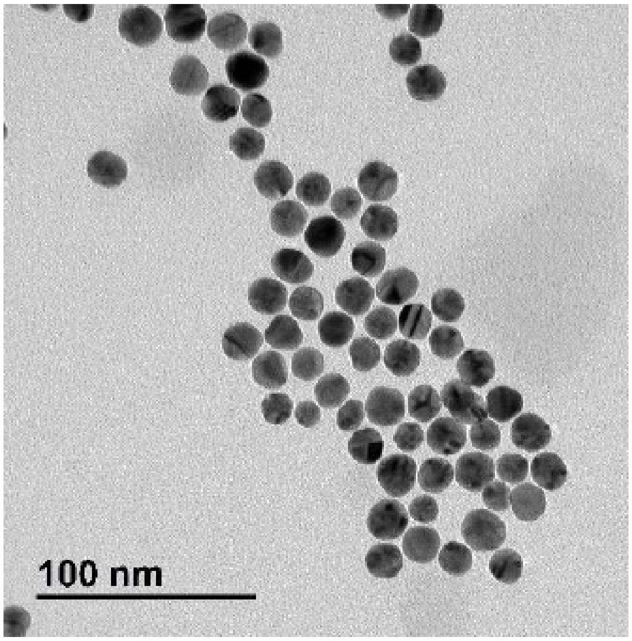


(a) TEM image of sphere n°2

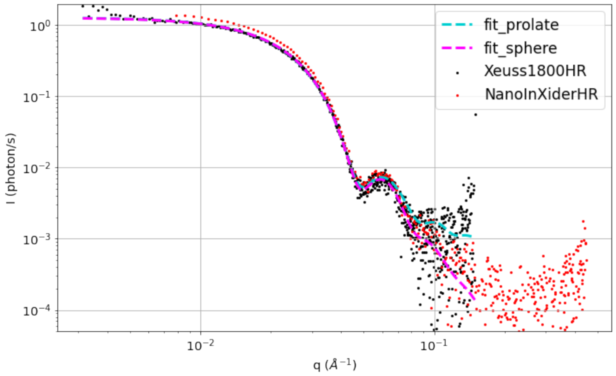


(b) SAXS curves of sphere n°2

Figure 3: TEM imaging, SAXS curve recorded on Xenocs devices and fits of the Xeuss1800HR curve for sample sphere n°2

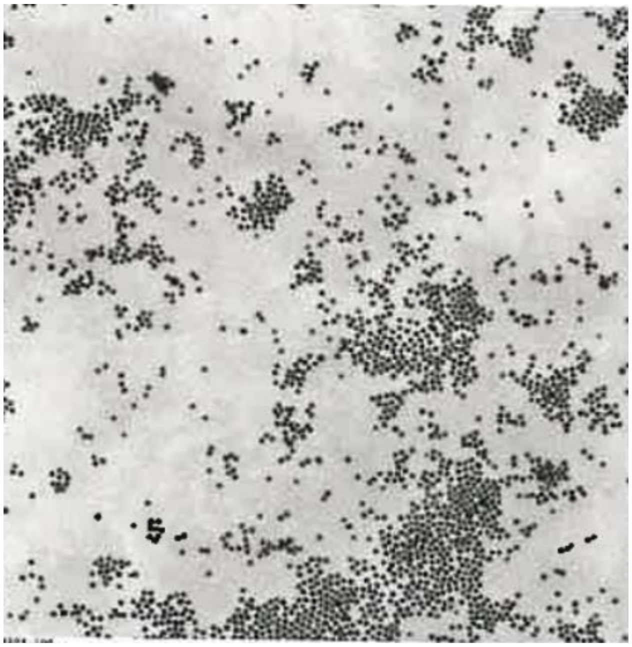

(a) TEM image of sphere n°3

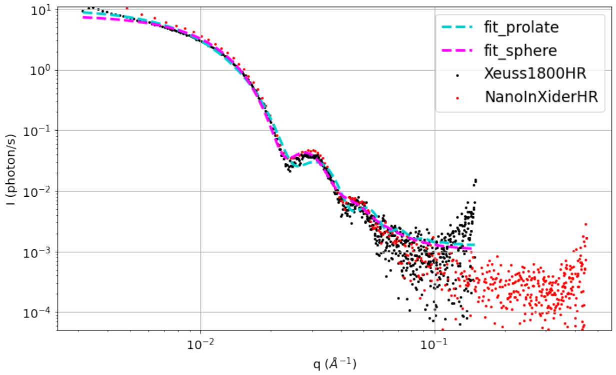


(b) SAXS curves of sphere n°3

Figure 4: TEM imaging, SAXS curve recorded on Xenocs devices and fits of the Xeuss1800HR curve for sphere n°3

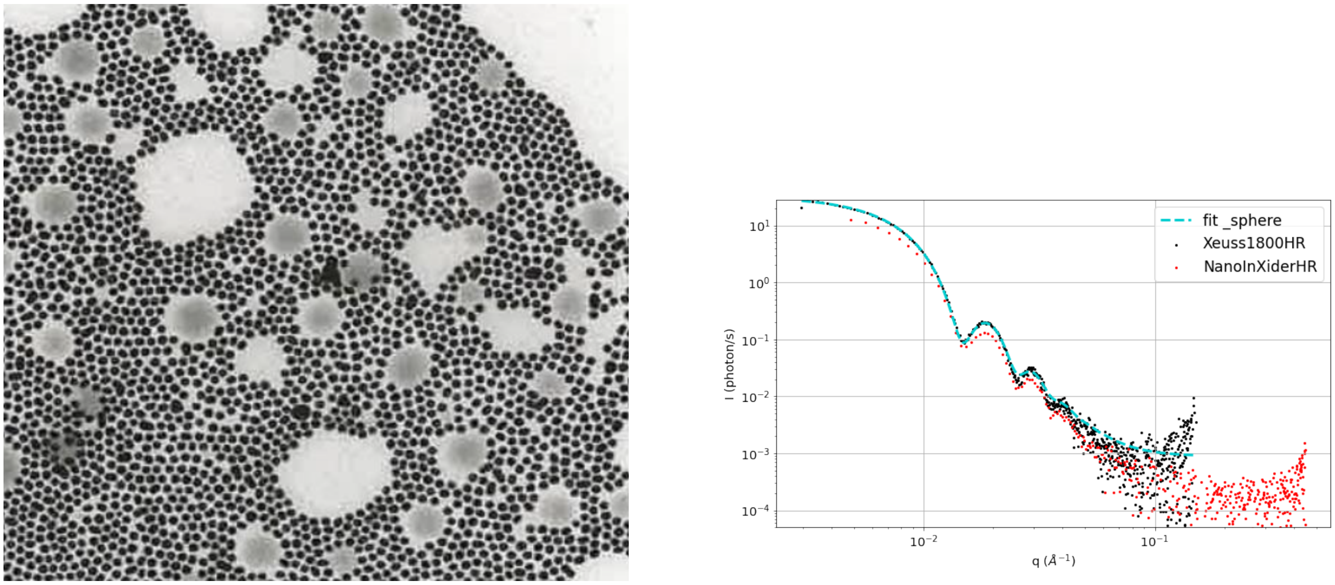


(a) TEM image of sphere n°4

(b) SAXS curves of sphere n°4

Figure 5: TEM imaging, SAXS curve recorded on Xenocs devices and fit of the Xeuss1800HR curve for sphere n°4

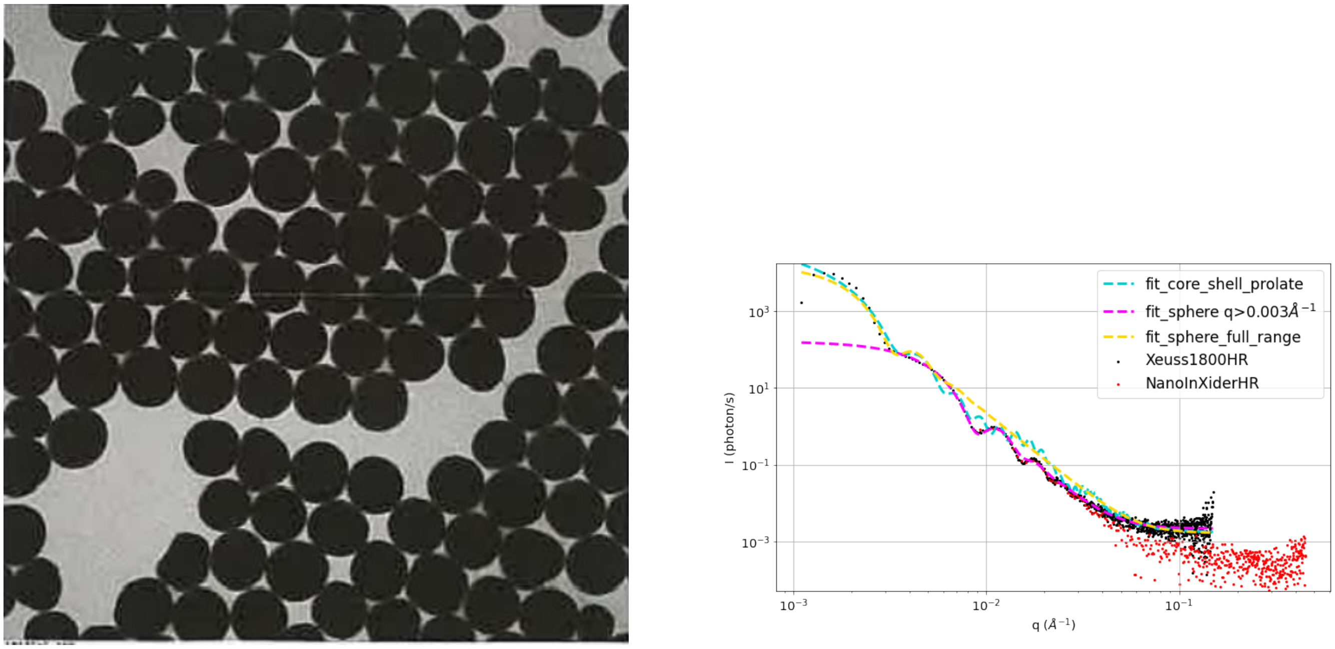


(a) TEM image of sphere n°5

(b) SAXS curves of sphere n°5

Figure 6: TEM imaging, SAXS curve recorded on Xenocs devices and fits of the Xeuss1800HR curve for sphere n°5

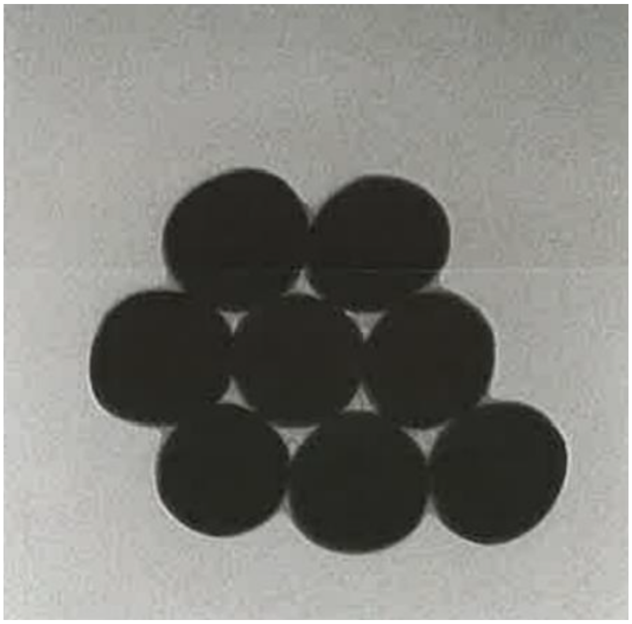

(a) TEM image of sphere n°6

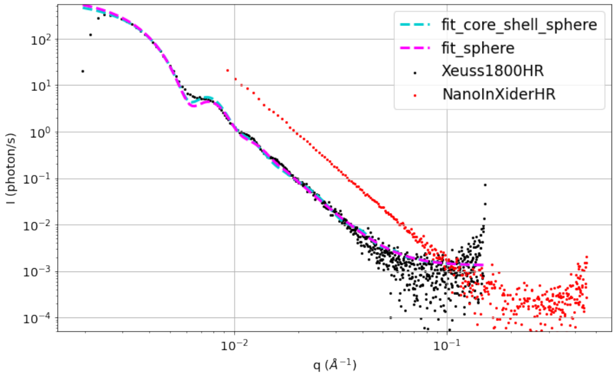


(b) SAXS curves of sphere n°6

Figure 7: TEM imaging, SAXS curve recorded on Xenocs devices and fits of the Xeuss1800HR curve for sphere n°6

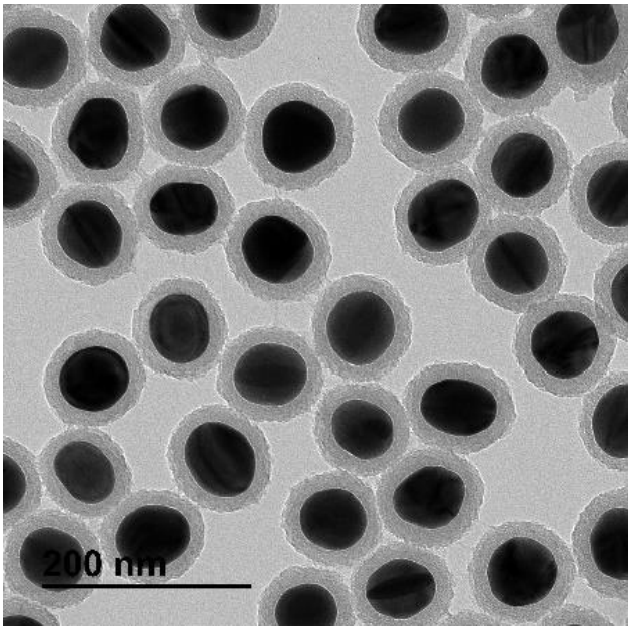


(a) TEM image of core shell sphere n°1

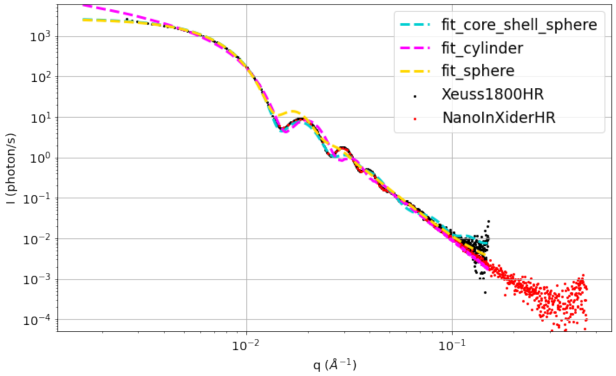


(b) SAXS curves of core shell sphere n°1

Figure 8: TEM imaging, SAXS curve recorded on Xenocs devices and fits of the Xeuss1800HR curve for core shell sphere n°1

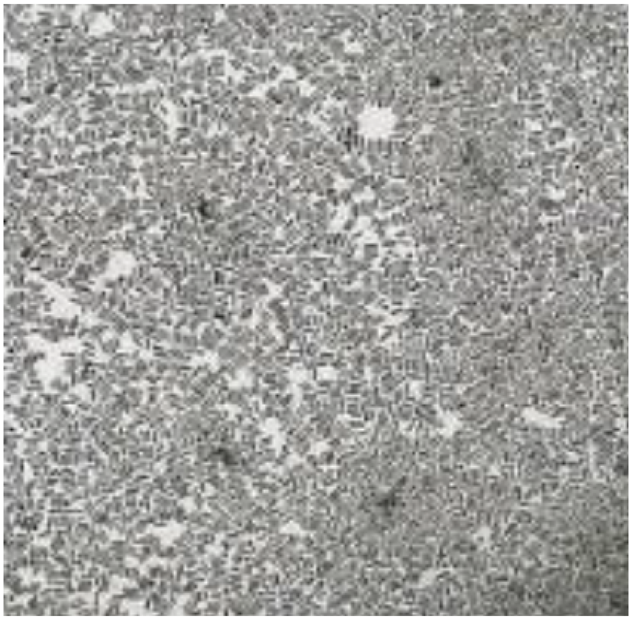

(a) TEM image of prolate n°1

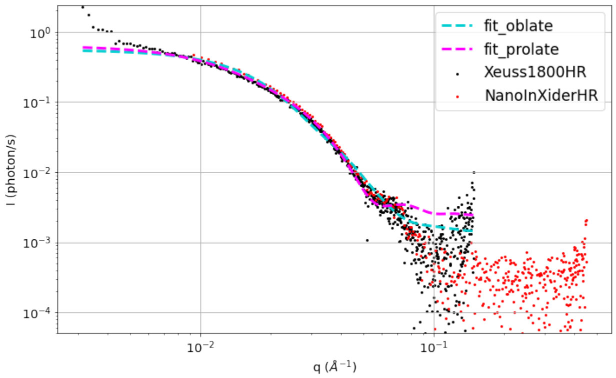


(b) SAXS curves of prolate n°1

Figure 9: TEM imaging, SAXS curve recorded on Xenocs devices and fits of the Xeuss1800HR curve for prolate n°1

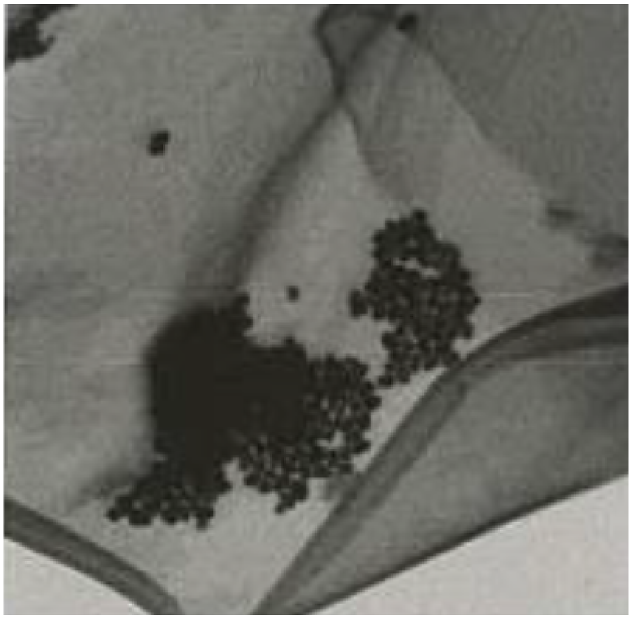

(a) TEM image of prolate n°2

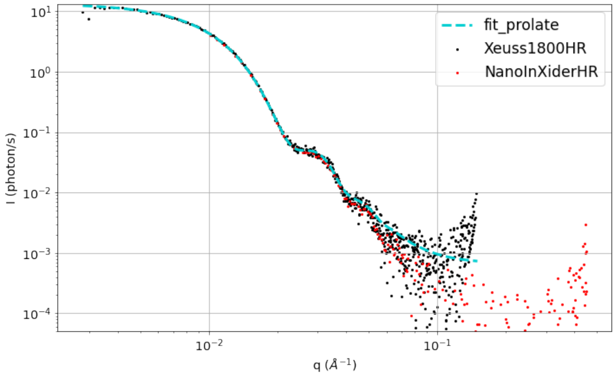


(b) SAXS curves of prolate n°2

Figure 10: TEM imaging, SAXS curve recorded on Xenocs devices and fit of the Xeuss1800HR curve for prolate n°2

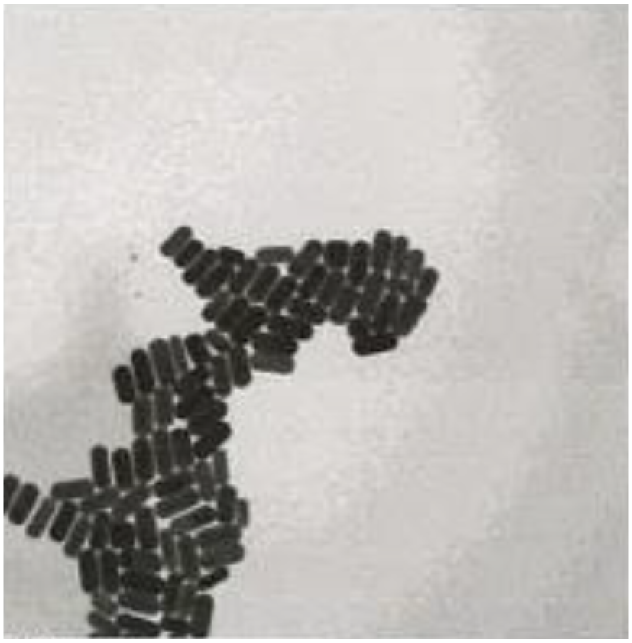

(a) TEM image of prolate n°3

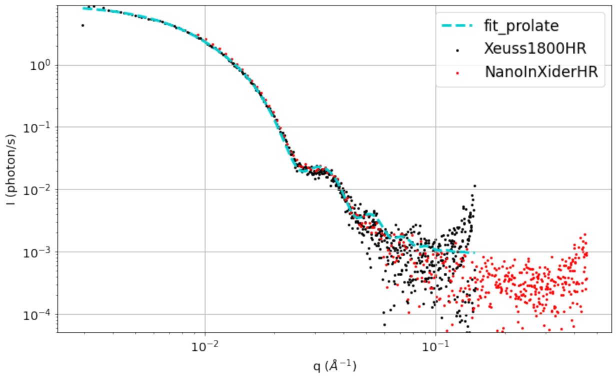


(b) SAXS curves of prolate n°3

Figure 11: TEM imaging, SAXS curve recorded on Xenocs devices and fit of the Xeuss1800HR curve for prolate n°3
